# Physiological movements during sleep in healthy adults across all ages: a video-polysomnographic analysis of non-codified movements reveals sex differences and distinct motor patterns

**DOI:** 10.1093/sleep/zsae138

**Published:** 2024-06-24

**Authors:** Angelica Montini, Giuseppe Loddo, Corrado Zenesini, Greta Mainieri, Luca Baldelli, Francesco Mignani, Susanna Mondini, Federica Provini

**Affiliations:** Department of Biomedical and NeuroMotor Sciences (DIBINEM), University of Bologna, Bologna, Italy; Department of Primary Care, Azienda AUSL Bologna, Bologna, Italy; IRCCS, Istituto delle Scienze Neurologiche di Bologna, UOC Clinica Neurologica Rete Metropolitana NEUROMET, Bellaria Hospital, Bologna, Italy; Department of Biomedical and NeuroMotor Sciences (DIBINEM), University of Bologna, Bologna, Italy; IRCCS, Istituto delle Scienze Neurologiche di Bologna, UOC Clinica Neurologica Rete Metropolitana NEUROMET, Bellaria Hospital, Bologna, Italy; Department of Biomedical and NeuroMotor Sciences (DIBINEM), University of Bologna, Bologna, Italy; IRCCS, Istituto delle Scienze Neurologiche di Bologna, UOC Clinica Neurologica Rete Metropolitana NEUROMET, Bellaria Hospital, Bologna, Italy; IRCCS, Istituto delle Scienze Neurologiche di Bologna, UOC Clinica Neurologica Rete Metropolitana NEUROMET, Bellaria Hospital, Bologna, Italy; IRCCS, Istituto delle Scienze Neurologiche di Bologna, UOC Clinica Neurologica Rete Metropolitana NEUROMET, Bellaria Hospital, Bologna, Italy; Department of Biomedical and NeuroMotor Sciences (DIBINEM), University of Bologna, Bologna, Italy; IRCCS, Istituto delle Scienze Neurologiche di Bologna, UOC Clinica Neurologica Rete Metropolitana NEUROMET, Bellaria Hospital, Bologna, Italy

**Keywords:** aging, behavioral sleep medicine, healthy participants, motor pattern, non-codified, periodic limb movements, sleep in women, sleep-related movements, sleep–wake physiology, video-polysomnography

## Abstract

**Study Objectives:**

To define sleep-related movements in healthy adults according to sex and age.

**Methods:**

Sleep-related movements from 50 video-polysomnography (vPSG) recordings of 27 men and 23 women, from 20 to 70 years old, were classified according to International classification of sleep disorders (ICSD-3-TR) and American Academy of Sleep Medicine (AASM) criteria (codified movements); the remaining movements (non-codified movements) were described according to type (elementary movements-EMs or complex movements-CMs), topography (focal, segmental, multifocal or generalized) and, if present, were assigned to motor patterns (MPs).

**Results:**

Of 4057 movements analyzed, 54.6% (2216/4057) were non-codified (1861 CMs, 355 EMs) and 1841 were codified. CMs were mainly generalized (70%) while EMs were multifocal (40%) or focal (30%). The median movement index (MI; movement/hour) was 11 and the median duration was 4 seconds. MI decreased from stages N1/REM > N2 > N3; men showed a higher MI. An MP was assigned to 2204 codified and non-codified movements, mainly *stretching* (50%) and *scratching* (30%). *Stretching* increased in REM sleep while *food-carrying behaviors* increased in N2. Men showed more *food-carrying behaviors*, *changes of body positions,* and *comfort movements* while *stretching* was more common in women. Younger participants exhibited more *food-carrying behaviors*, while *scratching* and *stretching* were more prevalent in the middle-aged group. Older participants showed more *changes in body positions* and *comfort movements*.

**Conclusions:**

In total, 54.6% of sleep-related movements in healthy participants were non-codified and characterized by motor sequences that can configure MPs. Our comprehensive classification method allows a detailed description of the physiological movements underlying differential motor control during sleep stages influenced by age and sex.

## Graphical Abstract

**Graphical Abstract:**
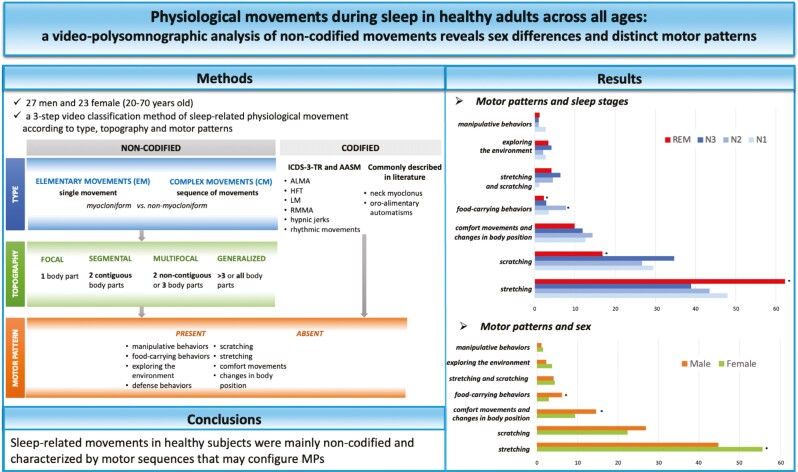

**Statement of Significance**

Not all movements observed during sleep in healthy participants correspond to the motor events defined in the International Classification of Sleep Disorders and American Academy of Sleep Medicine or commonly described in the literature. Our study proposed a three-step video classification method aimed at analyzing the heterogeneous sleep-related physiological movements combining movement type and topography with the identification of specific motor patterns. The latter may facilitate the differential diagnosis between physiological and pathological phenomena, especially in patients with nocturnal epilepsy or parasomnias where physiological movements could be misinterpreted as minor pathological events. We applied this method to 50 participants of different ages and found significant differences according to sleep stages, sex, and age. These findings highlight how these factors can influence motor control during sleep.

A wide range of motor events can be observed during normal sleep. The different expressions of motor activity depend on several elements within the sleep framework (sleep stage, delta power, and arousals) and on individual characteristics (i.e. age and habits) [1, 2]. Physiological movements during sleep have interested researchers since before the introduction of video-polysomnography (vPSG) through direct observation [3] or indirect signs, such as the electromyographic (EMG) signal, electroencephalographic (EEG) artifacts, and mechanical bedspring transducers [4–6]. By means of vPSG, the current gold standard for the detection and diagnosis of all sleep-related motor events, some physiological movements had already been characterized and included in the third edition of the International Classification of Sleep Disorders (ICSD-3-TR) and American Academy of Sleep Medicine (AASM) (i.e. limb movement- LM) [7, 8], while others were commonly reported in literature (i.e. neck myoclonus- NM) [9] or recently described as a new entity, such as large muscle group movements- LMMs [10]. The remaining sleep-related motor events are challenging to classify due to their multifaceted nature, thus there have been only a few attempts to categorize them in detail. Stefani et al. [11] proposed a classification based on movement complexity and the involvement of the different body parts while Brás et al. [12] focused on the motor patterns (MPs) associated with spontaneous motor arousals. The latter are sequences of movements, involving recurrent body areas probably produced by the activation during arousals of central pattern generators (CPGs) [13, 14]. The MPs range from simple, phylogenetically conserved automatic movements, such as swallowing, to more complex ones that resemble wakeful activities, such as head orientation. Physiological MPs were originally observed as incidental findings in vPSG recordings of patients with nocturnal epilepsy and NREM parasomnias but their recognition also in the healthy control participants suggested an intrinsic rather than a pathological sleep phenomenon [15–17]. This observation complicates the differential diagnosis between physiological and pathological motor events, as the boundaries between the two are still blurred in some cases. Thus, although many efforts have been made to describe and quantify movements during sleep in healthy individuals [18], the controversies in this field have not been fully resolved and a unified classification encompassing all features is currently lacking.

The aim of this study was to describe and quantify all physiological movements during sleep by combining their types, semiology, and topography with the identification of specific MPs through detailed video analysis. We assess this comprehensive classification method in different age groups to investigate possible differences depending on age, sex, and sleep stages.

## Methods

### Participants

Fifty healthy participants (10 for each decade, from 20 to 70 years of age) were recruited from a random population of caregivers of patients who were consecutively referred to Bellaria Hospital between 2017 and 2022.

After a first screening interview by a hospital neurologist, each healthy volunteer underwent a structured interview with a neurologist expert in sleep medicine.

The main inclusion criteria were whether participants perceived their sleep as restorative and Epworth Sleepiness Scale (ESS) score < 10. Exclusion criteria included the presence of any sleep disorder according to the ICSD-3-TR [7] as well as other neurological, psychiatric, cardiac, pulmonary, renal, hepatic, metabolic or oncological diseases, pregnancy, the use of psychoactive medication, regular alcohol and high caffeine consumption.

The study was approved by our local Ethical Committee (Code CE: 17176). The consent of healthy participants was obtained in agreement with the Convention of Helsinki.

### Video-polysomnography

All participants underwent an in-lab or home 24-hour nocturnal home-vPSG (XLTEK Trex HD, Natus Medical Incorporated®, video-camera Handycam HDR-CX700, Sony, 12.3 Megapixel resolution). All participants were instructed to keep their habitual routine on the day preceding the exam. During the night of the recording, participants had to sleep without blankets to allow the full detection of all movements. Video-polysomnography (vPSG) recordings included 3 EEG channels (frontal, central, and occipital), electrocardiogram, electro-oculogram, chin and both anterior tibialis electromyography, thoracoabdominal respirogram, and synchronized audio–video recording. Sleep stages were scored in 30-second epochs according to the AASM criteria [8].

### Movement identification and descriptors

All visible sleep-related movements were identified and classified according to type, topography and were eventually assigned to a MP (see below in the section “Movement Classification”) by a sleep medicine trainee (A.M.); the MP was reviewed by two sleep specialists (F.P., G.L.) and any disagreements were resolved through consensus at the time. To identify the movements, the time-synchronized video was analyzed if there was an increase in EMG activity with a duration of >100 milliseconds on the mentalis or limbs muscles channels [11] and/or a movement artifact on at least two channels [10]. We excluded movements arising from wake after sleep onset (WASO). The duration of the movements was calculated as the time between the exact onset and offset observed in the video. Video analysis was extended to at least 3 seconds mini-epoch before the motor event to determine the exact onset of the movement and the presence of possible external (i.e. ambient noise) or internal triggers (i.e. deep inspiration and cough). Two movements were considered separate if there was an interruption in the motor sequence lasting longer than 1 second [10] and the EEG between movements corresponded to a sleep stage.

For each movement, the temporal appearance during the night and the sleep stage of onset [10] were provided as descriptors.

### Movement classification

**Figure 1.**
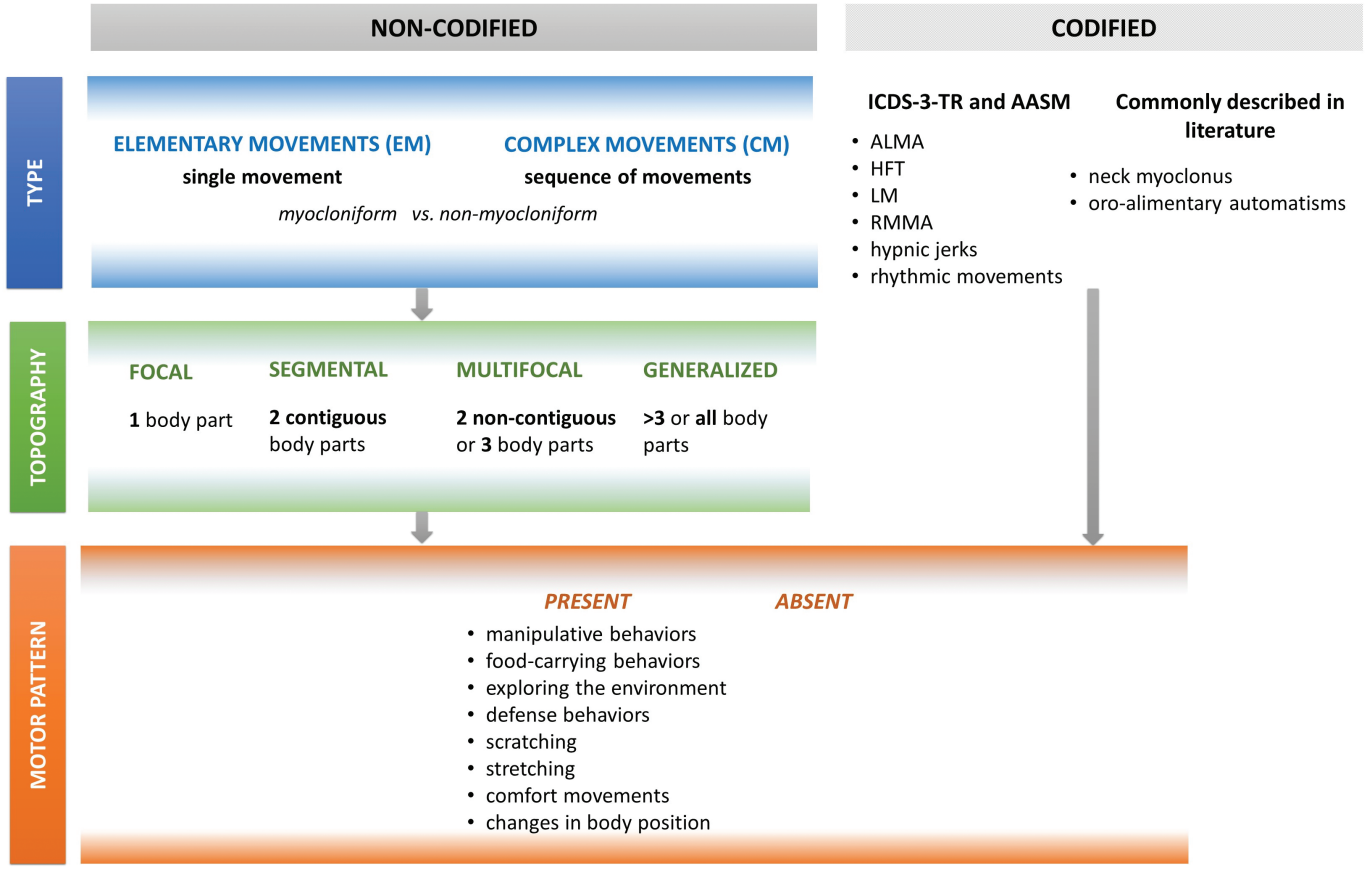
The three-step classification system for sleep-related movements in healthy participants, based on type, topography, and the identification of potential motor patterns (MPs). The movements analysis was conducted using a three-step method. In the first step, movements were categorized by type. Movements that did meet the criteria of International Classification of Sleep Disorders (ICSD-3-TR) [7] or the American Academy of Sleep Medicine (AASM) manual for the Scoring of Sleep and Associated Events [8] were commonly described in the literature [9, 11] were classified as codified. Codified Movements include limb movements associated with the EMG activity consistent with alternating leg muscle activation (ALMA), hypnagogic foot tremor (HFT), or leg movement (LM). Chewing-like movements associated with rhythmic masticatory muscle activity (RMMA) on the chin channel and/or only isolated oro-alimentary automatisms-OAs (including stereotyped movements involving the lower part of the face such as yawning, coughing, chewing, swallowing, tongue clicking, licking the lips and mouth opening/closing without the required EMG criteria for RMMA) were also included into the codified movements. The remaining non-codified movements were classified into elementary movements (EMs) and complex movements (CMs), depending on whether it was a single movement or a sequence of movements. In addition, the non-codified movements were divided into myocloniform and non-myocloniform, whether they resulted from a sudden muscle contraction or not. In the second step, EMs and CMs were classified by the number of body parts involved and categorized as focal, segmental, multifocal, or generalized. CMs topography (as they consisted of a sequence of movements) was analyzed both at the onset (T0) and at the end of the entire motor event (T1). The third step involved a comprehensive review of all movements to assign them to specific motor patterns (MPs). ALMA, alternating leg muscle activation; HFT, hypnagogic foot tremor; LM, limb movement; PLMS, periodic limb movements in sleep; RMMA, rhythmic masticatory muscle activity.

A) Movements were classified according to (A) type and (B) topography (adapting a previous classification proposed by Stefany et al. [11]); additionally, if present, they were assigned to (C) a MP (Figure 1). Since the type and topography of the “codified movements” are well-known, we have described these two aspects (A and B) only for the “non-codified movements,” while for the recognition of MPs (C), we have included all identified movements to ensure that no movements are omitted. Movements corresponding to those listed in the ICSD-3-TR [7] and the AASM manuals [8] or commonly described and accepted in the literature (NM and oro-alimentary automatisms-OAs) [9, 11] were classified as “codified movements.” The remaining motor events, the “non-codified” movements, were divided into elementary movements (EMs) and complex movements (CMs). The former were simple motions involving one or more body parts simultaneously, while the latter corresponded to a sequence of movements, eventually evolving into organized actions. The EMs and CMs were further characterized as myocloniform and non-myocloniform, respectively, if they were the results of a sudden, brief, shock-like muscle contraction or not.
B) Non-codified movements were defined as focal, segmental, multifocal, or generalized according to the number of body parts involved, considering five regions (head, upper trunk, lower trunk, arms, and legs). *Focal* movements involved only 1 body part, *segmental* 2 contiguous body parts, *multifocal* 2 noncontiguous or 3 parts (contiguous or not); *generalized* movements involved more than 3 noncontiguous body parts. As the CMs consisted of motor sequences, they were analyzed both at the beginning (T0) and at the end of the movement (T1).
C) Codified and non-codified movements were reviewed and eventually assigned to specific MPs. The latter were constituted by the combination of simple movements into integrated behaviors (such as *exploring the environment, defense behaviors, scratching, stretching, comfort movements, manipulative behaviors,* and *food-carrying behaviors*) or simple changes in body position associated or not with vocalizations. *Exploring the environment* pattern was represented by the opening of the eyes and/or a slow head rotation (“orientation”). *Defense behaviors* were represented by a jerky flexion of the arm, bringing the hand to the face as if to protect it. *Manipulative behaviors* consisted of fine hand movements as if to manipulate something, in isolation or preceded by arm flexion to locate objects. We chose the term *food-carrying behaviors* because the association of OAs (chewing/tongue-snapping/swallowing) with arm or foot movements with or without head movements, might resemble animal behavior [19], where preparatory actions to assess the distance to the target often precede food intake [20]. The *food-carrying behaviors* of MPs were also observed in codified movements when an LM was associated with OAs.

### Statistical analysis

In the descriptive analysis, the data were presented as the median and interquartile range (IQR) for continuous variables and with absolute (*n*) and relative frequency (%) for categorical variables. The movement index (MI) was defined as the sum of the number of movements divided by the hours of sleep (movements *per* hour) and was stratified by temporal distribution during the night (dividing the night into three parts), sleep stages, sex, and age of the participants. The Kruskal–Wallis test for continuous variables or Chi-square test for categorical variables were used for the group comparisons. Spearman’s Rho coefficients were used to evaluate the correlation between variables. The *p*-value was corrected for multiple comparisons (Bonferroni correction) and *p*-value < .05 was considered significant. Statistical analysis was performed using Stata SE 14.2.

## Results

### Participants and sleep macrostructure

We analyzed 50 vPSG of healthy participants (27 M, 23 F) with a median BMI of 23.5 (IQR 21.9–26.6). Participants were divided into five age groups: 20–29 years (four women); 30–39 years (five women); 40–49 years (four women); 50–59 years (six women); and 60–70 years (four women). The median age was 43 years (IQR 30–57). Fourteen patients presented with snoring and eight of them had also associated respiratory events, with a median apnea–hypopnea index of 1.3 (IQR 0.7–2.8) (Table 1).

**Table 1.** Sleep Macrostructure Parameters

Sleep parameters	Total	20–29 years	30–39 years	40–49 years	50–59 years	60–70 years
TTS (min)	395.0 (362.5–435.0)	388.0 (362.0–409.0)	408.0 (321.0–475.0)	409.0 (377.0–436.0)	394.3 (340.5–409.5)	392.5 (369.5–425.0)
SL (min)	6.0 (3.8–10.2)	11.0 (5.9–15.0)	6.9 (3.4–15.0)	4.1 (2.0–4.8)	6.1 (4.2–8.9)	6.5 (2.9–9.2)
REM L (min)	70.0 (59.0–83.0)	88.5 (63.5–132.0)	72.0 (66.0–83.0)	64.0 (53.0–74.5)	75.3 (66.0–82.0)	59.3 (42.0–69.5)
SE (min)	90.7 (85.6–93.6)	90.9 (88.4–93.6)	91.0 (86.6–95.0)	92.8 (91.0–96.4)	87.0 (81.2–93.0)	87.9 (85.0–91.3)
N1 (%)	6.0 (4.0–9.4)	6.3 (4.1–11.7)	4.0 (3.2–6.0)	5.5 (3.7–8.4)	5.8 (4.0–7.3)	12.0 (7.7–19.4)
N2 (%)	44.9 (37.6–50.4)	45.5 (37.6–50.7)	44.5 (39.4–54.1)	45.7 (44.3–51.5)	47.2 (39.1–49.6)	37.6 (32.4–38.2)
N3 (%)	26.3 (20.8–29.9)	27.5 (23.1–29.5)	26.2 (20.0–35.1)	27.3 (21.0–29.0)	27.3 (21.0–29.0)	28.8 (20.8–29.9)
REM *(%)*	21.5 (17.3–26.0)	17.4 (15.3–25.0)	21.1 (13.0–24.0)	24.5 (19.1–27.3)	22.7 (17.7–28.0)	21.9 (16.9–23.7)
PLMI^*AAMS*^ [8]	1.3 (0.5–8.6)	0.7 (0–4.8)	1.6 (0.8–5.4)	0.7 (0.6–7.3)	0.7 (0–1.6)	9.7 (3.2–24.7)
PLMI^*WASM*^ [39]	0.9 (0–6.9)	0.6 (0–2.7)	1.2 (0.1–4.8)	0.3 (0–6.5)	0.4 (0–1.6)	8.9 (2.9–24.7)

Values are presented as the median and interquartile range (IQR). TTS, total sleep time; SL, sleep latency; REM L, rapid eye movement (REM) sleep latency; SE, sleep efficiency; PLMI, periodic limb movements (PLM) index.

### Sleep-related movement analysis

#### Type of movements.

A total of 4057 movements were analyzed, 3488 (86%) spontaneous and 569 (14%) evoked (398, 70%, by an internal and 171, 30%, by an external stimulus). A proportion of 1841 (45,4%) movements fulfilled the ICSD-3-TR [7] or AASM [8] criteria or were commonly reported in the literature (*codified movements*). The remaining movements (*non-codified movements*) consisted of 1861 (45.9%) *complex movements* (CMs) and 355 (8.7%) *elementary movements* (EMs). Among the *codified movements*, we identified 1464 (79.5%) leg movements (LM), 131 (7.1%) isolated OAs, 120 (6.5%) NM, 38 (2.1 %) rhythmic movements, 25 (1.4%) hypnic jerks, 24 (1.3%) hypnagogic foot tremor, 23 (1.2%) alternating leg muscle activation, and 16 (0.9%) chewing-like movements associated with rhythmic masticatory muscle activity. Additionally, we observed OAs at the beginning (in 198 movements; 4.9%) or within the motor sequence (in 465 movements; 11.5%).

**Figure 2.**
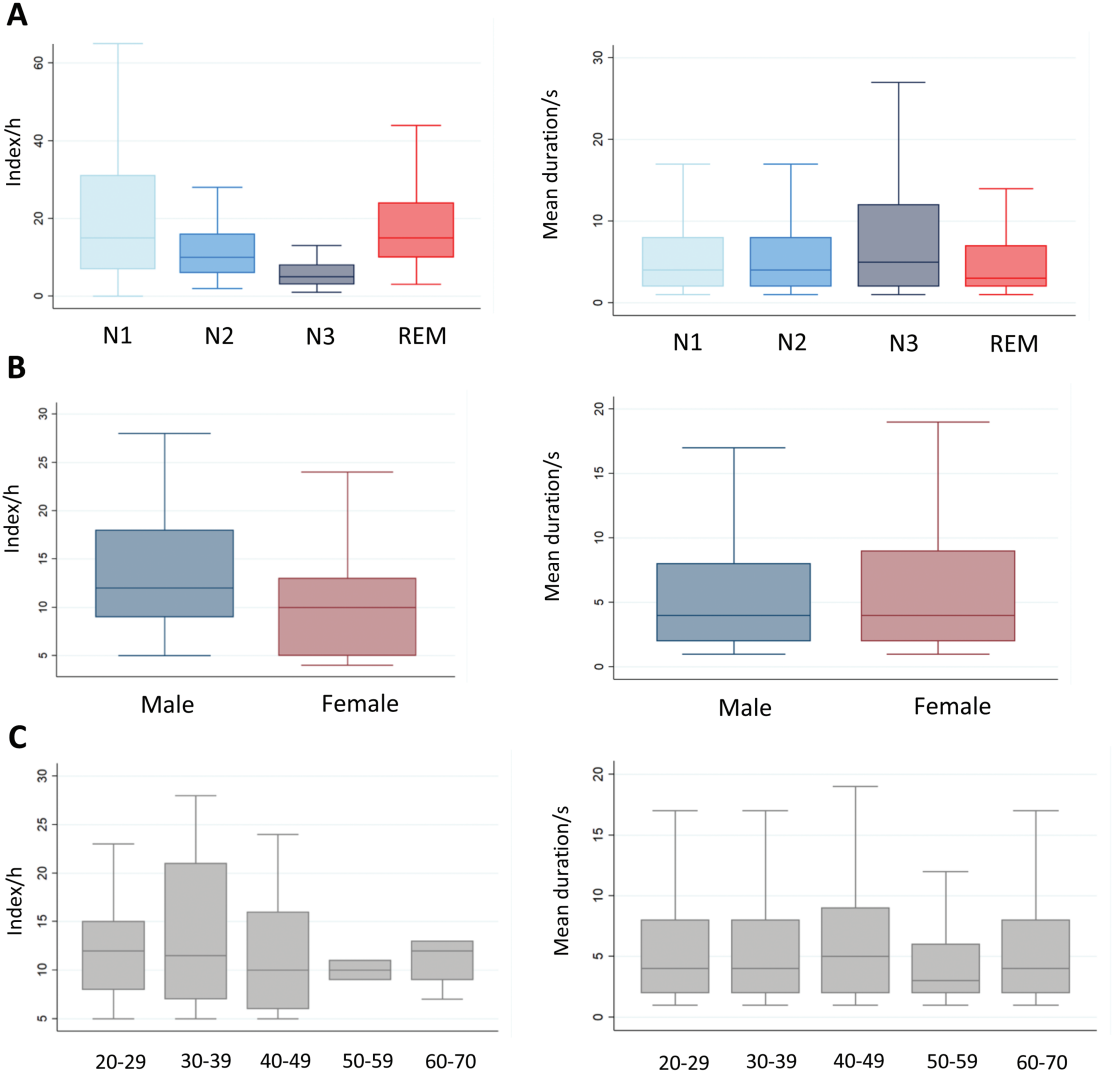
Distribution of movement index (MI) across the different sleep stages (A), sex (B) and age groups (C). Box and whiskers plot represent the MI indices (on the left) and median durations (on the right) across the different sleep stages (A), sex (B) and age groups (C). The central line represents the median, and the whiskers indicate the interquartile range.

The median total MI was 11 (IQR 8–15), with a homogeneous distribution throughout the three-thirds of the night (first tertile: median 10, IQR 7–14; second tertile: median 10, IQR 6–16 and third tertile: median 11, IQR 7–18). MI significantly decreased with the deepening of sleep, from N1 > N2 > N3 stages, to increase again during REM sleep (Table 2 and Figure 2A). The median duration of all movements was 4 seconds (IQR 2–8) and the movement duration was inversely proportional to sleep depth (N3 median duration: 5 seconds, IRQ 2–12; N1 vs. N3 *p* = .015, N2 vs. N3 *p* < .001), reaching its minimum during REM sleep (REM median duration: 3 seconds, IRQ 2–7; REM vs. N1/N2/N3 *p* < .001).

**Table 2.** Movement Indices (MI) According to Type and Topography Across the Different Sleep Stages

Movement	Total	N1	N2	N3	REM	*P*-value
**CODIFIED and NON-CODIFIED**	11 (8–15)	15 (7–31)	10 (6–16)	5 (3–8)	15 (10–24)	*N1 vs. N2 = .001* ** *N1 vs. N3 < .001* *** N2 vs. N3 < .001*** N2 vs. REM = .001** N3 vs. REM < .001***
**CODIFIED**	5 (2–7)	5 (0–17)	4 (1–8)	1 (0–3)	6 (3–9)	*N1 vs. N2 = .032* * *N1 vs. N3 < .001* *** *N2 vs. N3 < .001* *** *N2 vs. REM = .001* ** N3 vs. REM < .001***
**NON-CODIFIED**	6 (4–8)	8 (4–15)	6 (4–9)	3 (2–5)	8 (4–13)	*N1 vs. N2 = .023* * N1 vs. N3 < .001*** N2 vs. N3 < .001*** N2 vs. REM = .007** N3 vs. REM < .001***
***Elementary***	1 (0–2)	0 (0–3)	1 (0–1)	0 (0–1)	1 (0–3)	N2 vs. REM = .032* *N3 vs. REM* < .001***
* Myocloniform*	0 (0–0)	0 (0–0)	0 (0–0)	0 (0–0)	0 (0–1)	*N1 vs. REM = .001* ** *N2 vs. REM = .004* ** *N3 vs. REM = .010* *
* Non-myocloniform*	1 (0–1)	0 (0–3)	1 (0–1)	0 (0–1)	1 (0–2)	N1 vs. N3 = .034* N2 vs. N3 = .014* N3 vs. REM < .001***
***Complex***	5 (4–7)	7 (4–12)	5 (3–7)	3 (2–4)	6 (4–11)	*N1 vs. N2 = .016* * *N1 vs. N3 < .001* *** *N2 vs. N3 < .001* *** *N2 vs. REM = .029* * *N3 vs. REM < .001* ***
* Myocloniform*	1 (0–1)	0 (0–0)	0 (0–1)	0 (0–1)	1 (0–2)	*N1 vs. REM = .001* ** *N2 vs. REM < .001* *** *N3 vs. REM < .001* ***
* Non-myocloniform*	5 (3–6)	6 (4–12)	5 (3–6)	2 (1–4)	5 (3–9)	*N1 vs. N2 = .001* ** *N1 vs. N3 < .001* *** *N2 vs. N3 < .001* *** *N3 vs. REM < .001* ***
**Focal**	0 (0–1)	0 (0–2)	0 (0–1)	0 (0–0)	0 (0–1)	n.s.
**Segmental**	0 (0–0)	0 (0–0)	0 (0–0)	0 (0–0)	0 (0–0)	n.s.
**Multifocal**	1 (0–2)	2 (0–4)	1 (0–2)	1 (0–1)	2 (1–3)	*N1 vs. N3 = .011* * *N2 vs. N3 = .022* * *N3 vs. REM = .007* **
**Generalized**	4 (2–5)	4 (2–8)	3 (2–6)	2 (1–3)	5 (3–9)	*N1 vs. N3 < .001* *** *N2 vs. N3 < .001* *** *N2 vs. REM = .007* ** *N3 vs. REM < .001* ***

Values are presented as the median and interquartile range (IQR). The latter are presented by type (elementary and complex), subtype (myocloniform and non-myocloniform), and topography of the entire motor sequence (focal, segmental, multifocal, and generalized). Significant *p*-value after post hoc Bonferroni correction are presented; n.s. = non-significant (*p*-value > .05). Italics* represent significant difference (*p* < .05). * means *p*-value < .05, ** means < .01 and *** means < .001.

In comparison to females, males showed a significantly higher MI, particularly in the first third of the night (male/female MI = 12.5/7; *p* = .002) and shorter movement duration (*p* = .030; Table 3 and Figure 2B). Age was not associated with significant differences in MI, whereas movement duration significantly declined in the 50–59 year age group (median duration in 50–59 age group: 3 seconds, IRQ 2–6; 20–29 vs. 50–59 < 0.001, 30-39 vs. 50–59 = 0.002, 40–49 vs. 50–59 < 0.001, 60–70 vs. 50–59 = 0.001; Table 4 and Figure 2C*).* Women had a higher number of non-codified movements compared to men. EMs and myocloniform movements showed a significant increase during REM sleep. CMs with myocloniform onset were elevated in young adults and peaked in the 30–39 age group. Isolated OAs showed no significant differences in their MI across sleep stages, sex, and age.

**Table 3.** Movement Indices (MI) According to Type and Topography in Women and Men

Movement	Women	Men	*P*-value
*Codified and non-codified*	10(5–13)	11.5(9–17.5)	.044*
*Codified*	3(2–6)	5(3–8.5)	.039*
*Non-codified*	6(3–8)	7(5.5–8.5)	*.18*
*Elementary*	1(0–2)	1(0–2)	*0.56*
* Myocloniform*	0(0–0)	0(0–0)	*.50*
* Non-myocloniform*	0(0–1)	1(0–1)	*.50*
** ** *Complex*	5(3–6)	6(4–7)	*.13*
* Myocloniform*	1(0–1)	1(0–1)	*.92*
* Non-myocloniform*	4(2–5)	5(3–6.5)	*.12*
* Focal*	0(0–0)	0(0–1)	*.22*
* Segmental*	0(0–1)	0(0–0)	*.082*
* Multifocal*	1(0–1)	1.5(1–3)	*.072*
* Generalized*	3(2–5)	4(3–5)	*.14*

Values are presented as the median and interquartile range (IQR). The latter are presented by type (elementary and complex), subtype (myocloniform and non-myocloniform), and topography of the entire motor sequence (focal, segmental, multifocal, and generalized). Significant *p*-value after post hoc Bonferroni correction are presented; italics* represent significant difference (*p* < .05). * means *p*-value < .05, ** means < .01 and *** means < .001.

**Table 4.** Movement Indices (MI) According to Type and Topography in the Different Age Groups

Movement	20–29 years	30–39 years	40–49 years	50–59 years	60–69 years	*P*-value
*Codified and non-codified*	12(8–15)	11.5(7–21)	10(6–16)	10(9–11)	11.5(8–12)	*.87*
*Codified*	3(2–5)	4(2–6)	2(1–7)	5.5(3–7)	6(4–8)	*.48*
*Non-codified*	8(6–9)	7(4–12)	7(5–7)	5(3–6)	6(4–7)	*.31*
*Elementary*	2(1–2)	1(0–3)	0.5(0–1)	1(0–2)	1(0–1)	*.25*
* Myocloniform*	0(0–1)	0(0–0)	0(0–0)	0(0–1)	0(0–0)	*.10*
* Non-myocloniform*	1(0–1)	0(0–3)	1(0–1)	0(0–1)	1(0–2)	*.44*
** ** *Complex*	6(4–7)	5(3–11)	6.5(5–7)	4.5(2–6)	5(4–6)	*.41*
* Myocloniform*	1(0–1)	2(1–2)^*^	0(0–1)	0.5(0–1)	0.5(0–1)	.015*
* Non-myocloniform*	5(4–7)	4(2–8)	6(4–7)	3(2–5)	5(3–5)	*.26*
* Focal*	1(0–2)^**^	0(0–0)	0(0–1)	0(0–1)	0(0–0)	.002**
* Segmental*	0(0–0)	0.5(0–1)	0(0–0)	0(0–0)	0(0–0)	*.26*
* Multifocal*	2(1–3)	1(1–1)	1.5(0–2)	1(0–2)	1(1–2)	*.77*
* Generalized*	4(2–4)	4.5(2–10)	5(4–5)	2.5(2–5)	3.5(2–5)	*.36*

Values are presented as the median and interquartile range (IQR). The latter are presented by type (elementary and complex), subtype (myocloniform and non-myocloniform), and topography of the entire motor sequence (focal, segmental, multifocal, and generalized). Significant *p*-value after post hoc Bonferroni correction are presented; italics* represent significant difference (*p* < .05). * means *p*-value < .05, ** means < .01 and *** means < .001.

#### Topography of non-codified movements.

Among the non-codified movements, 1387 movements (62.7%) were *generalized*, 588 (26.5%) *multifocal,* 125 (5.6%) *segmental,* and 116 (5.2%) *focal*. Movement topography across sleep stages, sex, and age groups are presented in Tables 2, 3, and 4, respectively.

**Figure 3.**
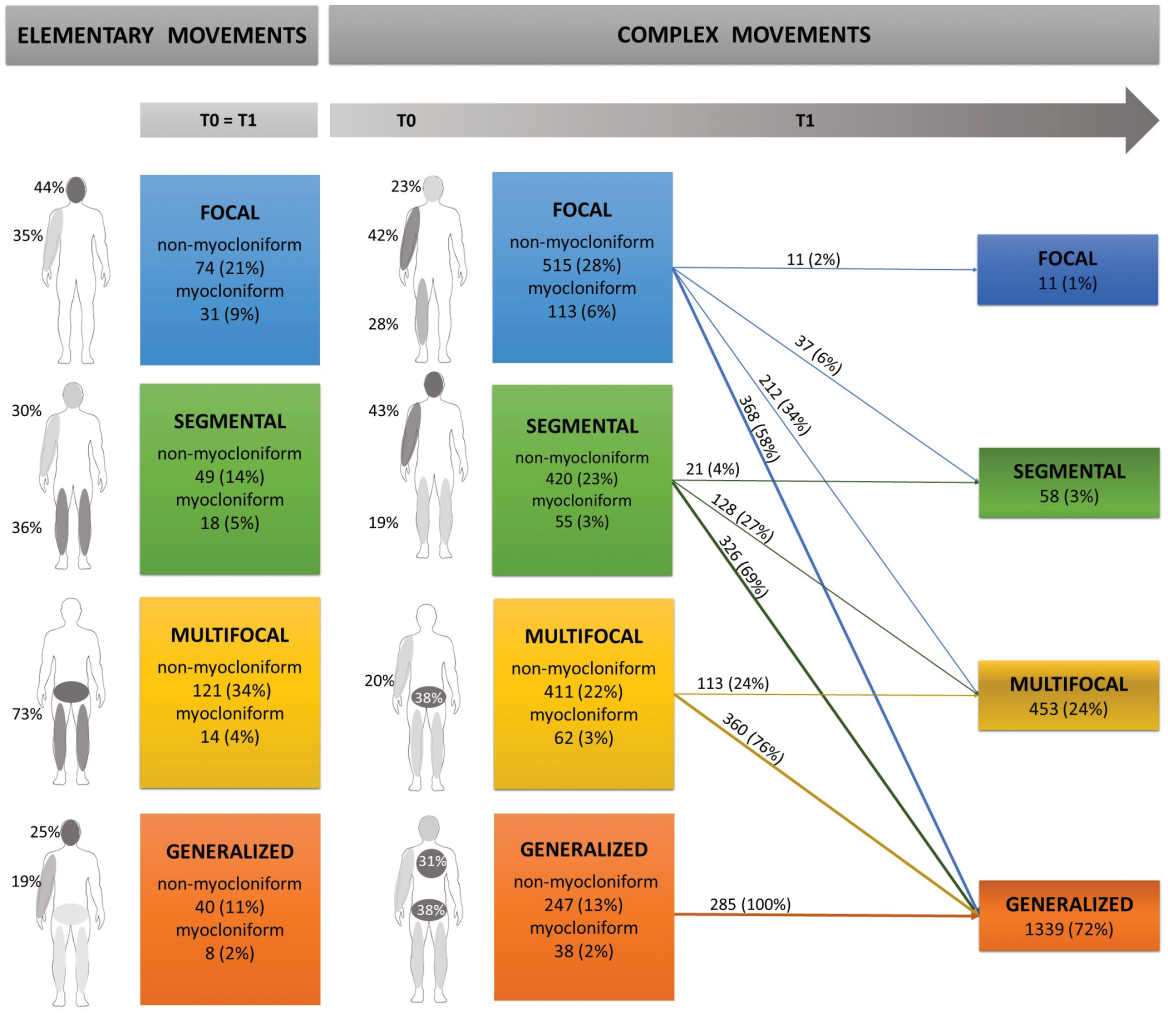
Topographical distribution of elementary (T0 = T1) and complex sleep-related movements (T0 and T1). The topography of the elementary movements-EMs (on the left) and the complex movements-CMs (on the right) is depicted both in relation to the body parts involved (grayscale of variable intensity based on the value of the frequency of involvement) and to the number of body regions involved (colored boxes). The topography is described both at the beginning (T0) and at the end (T1) of the motor sequence for the CMs with the respective percentages of motor sequence progression on the arrows. The data are presented in absolute numbers of movements and percentages. Percentages within the boxes refer to the total number of EMs (*n* = 355) and CMs (*n* = 1861) while those on the arrows correspond to the respective onset types; finally, the percentages on the body scheme reflect the number of movements involving the most commonly involved body areas (i.e. 46 EMs involved the head, corresponding to 44% of the focal EMs). Conventionally, in this illustrative scheme, the right arm is highlighted, but there is no significant difference between right and left when the first movement involves a limb. EMs were mostly multifocal movements involving the lower body regions (a combination of legs and lower trunk) or focal affecting the upper body (head or arm). EMs segmental involved legs or a combination of arm and head. Generalized EMs result from lower trunk movements with legs and head or ≥3 limbs movements. The onset (T0) of CMs motor was frequently a focal or segmental movement involving a combination of arm with head or legs. CMs began multifocal from the onset usually involving a combination of lower trunk with legs or three limbs. CMs were rarely generalized from the beginning and involved the lower trunk more than the upper trunk in combination with ≥3 limbs or legs and head.

EMs were, in descending order of prevalence*, multifocal* (135; 38%), *focal* (105; 30%), *segmental* (67; 19%), and *generalized* (48; 13%; Figure 3, on the left). The onset (T0) of CMs was focal in more than one-third of cases; then movements generalized or became multifocal (T1), particularly when they began as myocloniform (Figure 3, on the right).

#### Motor patterns.

MPs were identified in 2204 (54.3%) of the total sleep-related movements: 2151 (97.6%) were non-codified movements (1843 CMs, 308 EMs) and 53 (2.4%) were codified movements. The latter consisted of LM associated with OAs and were assigned to the *food-carrying behaviors* MP. The most frequently observed MPs were *stretching* (50%) and *scratching* (25%; Table 5) both of which were commonly observed at the beginning of a motor sequence (in nearly 85% of the cases), while all the other MPs were usually observed within the motor sequence. *Vocalizations* were rare (15 CMs), mostly occurring within the motor sequence and consisting of unintelligible moans and snorts.

**Table 5.** Age-Related Distribution of Specific Motor Patterns (MPs) at the Beginning of the Motor Sequence

Motor pattern	Total number	Median age	*P*-value
*Stretching (1)*	1081(49.0)	43(30–58)	*vs. 5 < .001* *** *vs. 2 < .001* ***
*Scratching (2)*	551(25.0)	41(28–54)	*vs. 1 < .001* ***
*Comfort movements (3) and change in body position (4)*	273(12.4)	46(30–56)	*vs. 5 = .002* **
*Food-carrying behaviors (5)*	107(4.9)	32(24–46)	*vs. 3 = .002* ** *vs. 1 < .001* ***
*Stretching (1) and scratching (2)*	93(4.2)	42(30–54)	*n.s.*
*Exploring the environment (6)*	63(2.9)	44(27–54)	*n.s.*
*Manipulative behaviors (7)*	27(1.2)	34(28–62)	*n.s.*

Categorical data were presented as absolute number and percentage, continuous data were presented as median and interquartile range (IQR). Significant *p*-values after post hoc Bonferroni correction are presented; n.s. = non-significant (*p*-value > .05). Italics* represent significant difference (*p* < .05). * means *p*-value < .05, ** means < .01 and *** means < .001.

**Figure 4.**
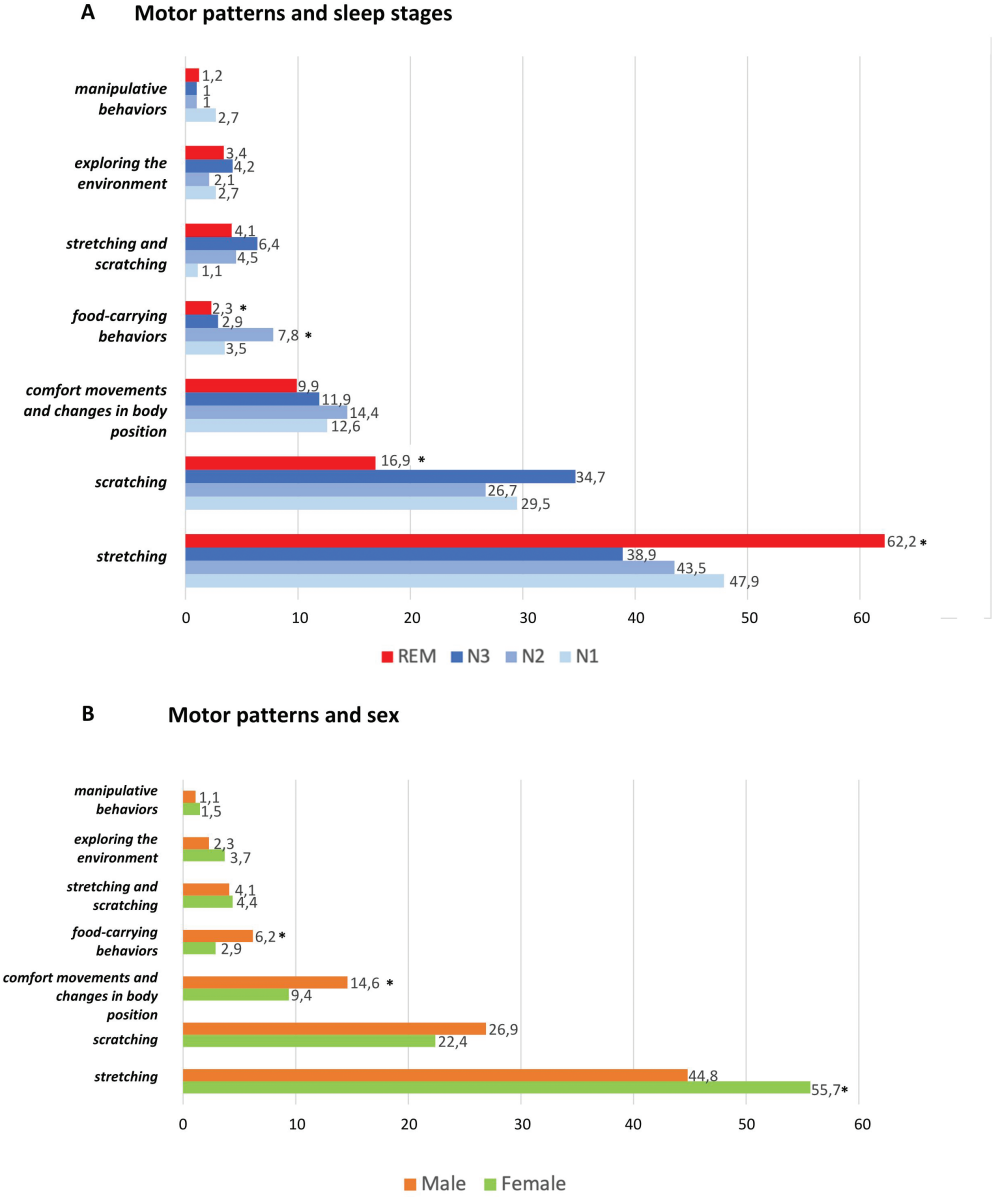
Distribution of specific sleep-related motor patterns (MPs) across the different sleep stages (A) and sex (B). Data were presented as percentage. Bold* represent significant difference (*p* < .05).

*Food-carrying behaviors* were more frequent (+25%) in N2 NREM stage while *stretching* increased (+8%) and *scratching* and *food-carrying behaviors* were reduced (−10% and −16%, respectively) in REM stage (Figure 4A). Exploring the environment MP was mostly observed in NREM stage (45; 67.2%) and, in one-fifth of the cases, it was evoked by triggers. One-third of the exploring the environment MPs began with the eye-opening whereas this MP appeared more frequently within the motor sequence (out of 93 CMs) while the participant maintained a neutral facial expression; exploring the environment MPs was rarely associated with a head lifting (15; 38.5%). Men showed a higher frequency of *food-carrying behaviors* (*p* = .003), *change of body position, and comfort movements* (*p* = .002), while women frequently showed *stretching* (*p* ≤ .001) and *exploring the environment* MPs (*p* = .35; Figure 4B). Younger participants (median 32 years) showed more *food-carrying behaviors;* in the middle age group (median 41 and 43 years, respectively) *scratching* and *stretching* were the most frequent MPs; older participants (median 46 years) showed more often *change of body position* and *comfort movements* (Table 5). In Supplementary Materials we include examples of the *manipulative behaviors*, *food-carrying behaviors* and *exploring the environment* MPs.

## Discussion

In our cohort, we observed that more than half of the sleep-related physiological movements could not be assigned to any specific classified motor event according to ICSD-3-TR [7] or AASM [8] criteria or any other movement commonly described in literature. The challenge in classifying these non-codified movements lies in their variability within and between participants throughout the night. To overcome this, our classification method builds upon previous attempts and integrates various movement aspects of increasing complexity. We began with a dichotomous distinction of movements based on type, categorizing them as either isolated (EMs) or organized into motor sequences (CMs). Then, we focused on topography which depends on the number of body areas involved. Finally, the recurrent combination of body areas led to the identification of specific MPs, which sometimes also have behavioral significance.

We found that most of the non-codified movements were CMs that typically started as focal movements, often involving the face or leg, and subsequently involved the whole body, particularly when they began as myocloniform. Movements occurred mainly in N1 and REM stages; as NREM sleep deepened, MI decreased significantly while duration increased in line with previous data from the literature [2, 11, 18, 21]. Males showed more movements than females as previously described [11, 18] and of shorter duration. Although the differences in MI between age groups did not reach statistical significance as in other studies [11, 18], MI is higher in the age groups at the extremes (<39 years and >60 years) potentially reflecting a mechanism of maturation [2, 22] and aging-related changes [23, 24] of the central inhibitory system.

In over half of the sleep-related movements, an MP was assigned: *stretching* and *scratching* were the most observed MPs, particularly at the beginning of the motor sequence. *Stretching,* which tended to be briefer and simpler, was frequently identified during REM sleep where movements are typically short-lasting and jerky [11, 12, 18, 25]. Otherwise, longer motor sequences were associated with better organized MPs, which often occurred during NREM sleep such as *food-carrying, scratching,* and *exploring the environment* MPs. Accordingly, in a cohort of 25 healthy young adults, Brás et al. [12] observed a higher prevalence of chewing in N2 stage, while *scratching* and exploratory behaviors were frequently observed during N3 stage.

In males, *comfort movements* and *changes of body position* prevailed along with *food-carrying behaviors,* which possibly might reflect their ethologically conserved role in procuring food. Conversely, women exhibit more *stretching* movements, which in animals represent a defensive behavior and exploration of peri-personal space, possibly to protect offspring from dangers [26, 27]. In fact, *stretching* might represent an information-gathering activity that allows us to mentally visualize the body schema during the night, converting the multiple inputs from receptors and, at the same time, identifying potentially dangerous environments [28].

In the middle-aged group, we found mainly *scratching* and *stretching,* which seem to have the common function of stress reduction in several animal species. *Scratching* rate in fact increases with sympathetic activation and during conflict in several animal species such as chimpanzees, long-tailed macaques, and baboons [29, 30]. *Scratching* during sleep is frequently observed in patients with epilepsy [15, 31–33] where it can be triggered or not by the ictal discharge. However, *scratching* in healthy participants appeared slower, less repetitive, and stereotyped compared to epileptic patients [15]. *Stretching* could reduce stress, as it does during the day [34] and may also be the result of a proprioceptive feedback mechanism that benefits the musculoskeletal system, particularly triggered during sleep by prolonged immobility.

Although no clear sexual behaviors were observed in the healthy participants, the *stretching* MP, when it involved the hips, could resemble the “pelvic thrusting” observed in motor episodes associated with epilepsy [35, 36] and sexsomnia [37]. During a seizure, however, the hip-swinging tends to be more pronounced and repetitive. Furthermore, when the *stretching* involves the limbs, it may resemble a tonic or dystonic posture, raising suspicion of an epileptic manifestation [38], especially if it occurs early in the movement sequence. However, physiological *stretching* is characterized by less sustained contractions and a more natural and symmetric posture. Otherwise, *exploring the environment* MP poses a challenge for differential diagnosis with the Disorders of Arousal episodes (DoA). However, in healthy participants, this MP occurred frequently in the middle-aged group, was more commonly evoked by triggers and occurred later in the motor sequence. When occurring early, it was not associated with trunk elevation, as if trying to get out of bed, or with expressions of confusion as observed in DoA episodes [16]. Finally, in older participants, there is an increased prevalence of the simplest MPs, *comfort movements* and *change of body positions*, which may be associated with a reduced cortical inhibition and movement organization that follows the course of cortical involution.

One strength of this study lies in our extensive effort to describe a large number of sleep-related physiological movements. To this end, we examined video data in detail even in the presence of artifacts and instructed participants to sleep without blankets. Our work is in line with a prior study that proposed a classification system for the non-codified physiological movements during sleep, but we expanded this classification by also incorporating the recognition of specific MPs. The latter could ease the differential diagnosis between physiological and pathological motor events occurring in patients with parasomnias and nocturnal epilepsy, particularly in the presence of minor motor events which are the most challenging. Furthermore, we had carefully selected a homogeneous group of healthy participants, balanced in terms of age and sex.

Our study has also some limitations to disclose. Small movements of the upper limbs and trunk might be lost due to the methodology chosen to identify movements. The sample consists of a single ethnic group, which could limit the generalizability of our results. Additionally, we only observed one night *per* participant, which prevents the assessment of inter-night differences or adaptation effects. However, many of our participants had undergone home-based vPSG, which allowed them to be in a familiar environment. It is important to note that we did not focus on facial expressions as the single camera was not always directed toward the face. Nonetheless, we did not observe overt facial expressions, unlike episodes of parasomnias and epilepsy.

## Conclusion

This is the first study that comprehensively classified the non-codified sleep-related movements in healthy participants, combining their type and topography with the recognition of specific MPs. The latter results from the observation of a recurring combination of different body parts that resemble behaviors that have been preserved throughout the phylogenetic scale. Thanks to this three-step approach, we were able to describe and quantify sleep-related movements that would otherwise have been neglected yet account for the majority of movements in healthy participants of all ages, particularly in the female population.

Compared to a previous study [11], of which we followed the nomenclature, we observed that most of the non-codified movements during sleep were complex, consisting of motor sequences with a focal onset and subsequently involving multiple body areas or becoming generalized. The topographic distribution also differs from that previous study [11], since we specifically focused on the non-codified movements.

The observed differences in movement features as a function of sleep stages, sex, and age in healthy adults suggest that sleep-related movements are controlled by different CNS generators.

In particular, the movement gate during sleep is related to sleep depth and CNS integrity, both of which vary with age. Younger individuals showed a higher rate of movements, mostly myocloniform, suggesting an ongoing maturation process. In middle age, there were fewer movements during sleep, especially stretching and scratching, which probably served to relieve stress and prolonged immobility. In older participants, reduced motor inhibition due to neurodegeneration could lead to simpler and non-myocloniform behavioral episodes, like comfort movements. The sex differences observed in sleep-related movements, although speculative due to the lack of data in the literature on this topic, appear to preserve behaviors that are ethologically and phylogenetically ingrained and may be related to evolutionary or hormonal differences, such as feeding behavior in males and defense against potential environmental threats in females.

The main strength of the study is the identification of specific MPs that might help clinicians by providing criteria for recognizing physiological movements during the nights of patients with sleep-related disorders. This becomes even more significant as home videos are gaining prominence, serving not merely as a screening tool. It also opens new windows in the field of research, where automatic video analysis of movements is emerging and has the potential to recognize and assess movement characteristics.

## Supplementary Material

Supplementary material is available at *SLEEP* online.

zsae138_suppl_Supplementary_Material

zsae138_suppl_Supplementary_Video

## Data Availability

The data underlying this article are available in the article and its online supplementary material. The data on which this article is based were provided with the patients’ consent.
