# Supplementary material for: Physiological movements during sleep in healthy adults across all ages: a video-polysomnographic analysis of non-codified movements reveals sex differences and distinct motor patterns
Source: Sleep. 2024 Jun 24;47(9):zsae138. doi: 10.1093/sleep/zsae138 (PMC11381566; doi:10.1093/sleep/zsae138)
Supplement: zsae138_suppl_Supplementary_Material [file zsae138_suppl_supplementary_material.docx]

**Physiological movements during sleep in healthy adults across all ages:**

**a video-polysomnographic analysis of non-codified movements reveals sex differences and distinct motor patterns**

Angelica Montini, MD; University of Bologna, Department of Biomedical and NeuroMotor Sciences (DIBINEM); [angelica.montini2@unibo.it](mailto:angelica.montini2@unibo.it).

Giuseppe Loddo, MD, PhD; Azienda AUSL Bologna, Department of Primary Care; [loddogiuseppe0@gmail.com](mailto:loddogiuseppe0@gmail.com)

Corrado Zenesini; IRCCS Istituto Delle Scienze Neurologiche di Bologna, Epidemiology and Biostatistics Unit; corrado.zenesini@isnb.it

Greta Mainieri, MD, PhD; University of Bologna, Department of Biomedical and NeuroMotor Sciences (DIBINEM); IRCCS Istituto Delle Scienze Neurologiche di Bologna, UOC Clinica Neurologica Rete Metropolitana NEUROMET, Bellaria Hospital; greta.mainieri2@unibo.it

Luca Baldelli, MD; University of Bologna, Department of Biomedical and NeuroMotor Sciences (DIBINEM); IRCCS Istituto Delle Scienze Neurologiche di Bologna, UOC Clinica Neurologica Rete Metropolitana NEUROMET, Bellaria Hospital; luca.baldelli4@unibo.it

Francesco Mignani; IRCCS Istituto Delle Scienze Neurologiche di Bologna, UOC Clinica Neurologica Rete Metropolitana NEUROMET, Bellaria Hospital; francesco.mignani7@unibo.it

Susanna Mondini, MD; IRCCS Istituto Delle Scienze Neurologiche di Bologna, UOC Clinica Neurologica Rete Metropolitana NEUROMET, Bellaria Hospital; susannamondini5@gmail.com

Federica Provini, MD, PhD; University of Bologna, Department of Biomedical and NeuroMotor Sciences (DIBINEM); IRCCS Istituto Delle Scienze Neurologiche di Bologna, UOC Clinica Neurologica Rete Metropolitana NEUROMET, Bellaria Hospital; [federica.provini@unibo.it](mailto:federica.provini@unibo.it)

***Video legend***

***Video 1. Samples of the three most commonly identified behavioural Motor Patterns (MP).***

**Exploring the environment MP** arousing from stage 3 NREM sleep: the patient opens her eyes, raises her head and left leg, looks around and scans the environment while making comfort and stretching movements with her legs.

**Manipulative MP** from stage 2 NREM sleep: the patient in a prone position raises his right arm, starts to touch the mattress with his eyes open, then grasps the polysomnography device, manipulates the various equipment components and then quickly raises his upper body while continuing the objects manipulations before returning to the prone position.

**Food-carrying MP**, starting from stage N2: the patient lies on his right side and begins to chew. Immediately afterwards, he slowly turns his head, and then the trunk, to the left until changing position to supine position while continuing to chew and repeatedly flexing both arms towards his face.
